# Antibacterial and anticancer properties of *Streptomyces microflavus* BA2 isolated from brackish waters

**DOI:** 10.1038/s41598-026-51609-5

**Published:** 2026-05-20

**Authors:** Basma M. Atallah, Ramadan El-domany, Hamdy E. Agwa, Eithar El-Mohsnawy

**Affiliations:** 1 Botany and Microbiology Department, Faculty of Science, Kafrelsheikh University, Kafr El-Sheikh, 33516 Egypt; 2 Microbiology and Immunology Department, Faculty of Pharmacy, Kafrelsheikh University, Kafr El-Sheikh, 33516 Egypt

**Keywords:** *Streptomyces microflavus*, Burullus lake, Antibiotic, GC-MS analysis, Anticancer, Biotechnology, Cancer, Drug discovery, Microbiology

## Abstract

The increasing incidence of cancer and the rapid emergence of antibiotic-resistant pathogens represent two of the greatest global health threats and necessitate safe, multifunctional agents. In the present study, the brackish water actinomycete *Streptomyces microflavus* BA2 was isolated from sediments of Lake Burullus (Egypt). Morphological and molecular analyses, including 16 S rRNA sequencing, confirmed its membership in the family Streptomycetaceae with 100% agreement to *S. microflavus* NRRL B-2156. The culture filtrate showed potent antibacterial activity against *Staphylococcus aureus*, *Klebsiella pneumoniae*, *Proteus mirabilis*, *Salmonella typhi* and *Escherichia coli*, with the aqueous diethyl ether fraction exhibiting the highest inhibition. GC-MS analyses identified seven bioactive compounds, including five fatty acids with antibacterial and anticancer properties, as well as phenolic derivatives. The diethyl ether extract showed moderate antitumor activity against HepG-2 liver cancer cells (IC₅₀ = 189.66 µg/ml), moderate antioxidant potential (IC₅₀ = 74.15 ± 3.27 mg/ml), and safe to moderate toxicity to normal WI-38 lung fibroblasts. These results underscore the high biomedical potential of *S. microflavus* BA2 as a promising source of natural agents for combating cancer and antibiotic resistance.

**Supplementary Information:**

The online version contains supplementary material available at 10.1038/s41598-026-51609-5.

## Introduction

There is an immediate need for novel antibiotics that are both effective and safe due to the increasing prevalence of antimicrobial resistance (AMR). Global public health and clinical outcomes are at risk due to multidrug-resistant (MDR) bacteria, viruses, and fungi, including *E. coli*, *Staphylococcus aureus*, and *Pseudomonas aeruginosa*^1^. Here, actinomycetes, and *Streptomyces* in particular, continue to play a pivotal role in the development of new antibiotics, with almost two-thirds of all antibiotics now in use coming from this microbial group^2^. But yields have been falling due to overexploitation of terrestrial resources, and known chemicals are being found again and again^3^. Because of this, there has been a change in focus toward looking for new actinomycetes in wild and harsh places. Marine and brackish ecosystems, in particular, are among these areas that have shown promise. Rare actinomycetes with various metabolisms and the ability to produce secondary metabolites with a wide range of structural diversity and biological activity have evolved in response to these stresses^4,5^. For instance, microbial bioprospecting has paid little attention to brackish environments, which provide a hybrid biological niche between freshwater and marine systems. Typically found in coastal lagoons, marshes, or estuaries where freshwater flows meet salty ocean currents, brackish ecosystems are known for their biological diversity. Adapted to nutrient flow, sediment fluctuation, and osmotic stress, these environments support a rich diversity of microbial communities. Rare actinomycetes with extraordinary biological capabilities may flourish in these environments. Diab et al.^6^ and Germa et al.^7^ found that actinomycetes isolated from brackish sediments had strong antioxidant, anticancer, enzymatic, and antibacterial properties, which suggests they might be used in biotechnology. The Mediterranean Sea’s coastal and brackish regions in northern Egypt are home to an exceptionally diverse array of microbes. From this area’s black sand beaches, Atallah et al.^8^ were able to extract *Streptomyces griseorubens* and *Streptomyces rochei*, which showed strong antibacterial action against MDR bacteria. A variety of bioactive chemicals, such as aromatic derivatives and fatty acids, were discovered via their gas chromatography-mass spectrometry (GC-MS) investigation. El-Mohsnawy et al.^9^ provided further evidence that these isolates are safe for the environment, implying that they might one day replace synthetic antimicrobials in aquaculture and other environmental contexts. Biologically active compounds such as alkaloids, phenols, fatty acids and macro-lactams exhibit significant antifungal, antibacterial and anticancer properties^4,5^. The metabolic characteristics and therapeutic potential of marine invertebrates are further impacted by the symbiotic interactions that these microbes often form with these creatures^10^. Negligible biomass production, complicated metabolite extraction techniques, and challenges with culture mean that marine and saltwater actinomycetes are seldom used in drug development programs, even though they have a lot of promise^11^. We are starting to break through these obstacles thanks to developments in bioinformatics and molecular biology. Rare actinomycetes and clusters of genes involved in biosynthesis have been identified thanks to technological advancements including genome mining, metagenomic analysis, and 16 S rRNA gene sequencing^2^. Pan et al.^5^ reported that these technologies have opened new paths for antibiotic discovery by revealing the existence of quiet or cryptic gene clusters. These clusters may be triggered by co-culturing, genetic manipulation, or synthetic biology approaches. Aside from its significance in medicine, aquatic actinomycetes also have positive effects on environment. They play an important role in environmental sustainability due to their capacity to break down pollutants, create natural pigments, and function as biological control agents^6,7^. Because of their two medicinal uses, they are perfect for integrated biotechnological projects. Here, the purpose of isolating and characterizing an actinomycete strain from northern Egypt’s brackish water is to find isolates that may provide safe and efficient antibacterial and anticancer chemicals. Present study aims to introduce ecofriendly, natural compounds capable of combating cancer and multidrug-resistant bacteria. It focuses on a biodiverse but underexplored region, with the goal of providing sustainable treatment alternatives.

## Results

### Identification of *Streptomyces microflavus *strain BA2

#### Morphological characteristics

**Fig. 1:**
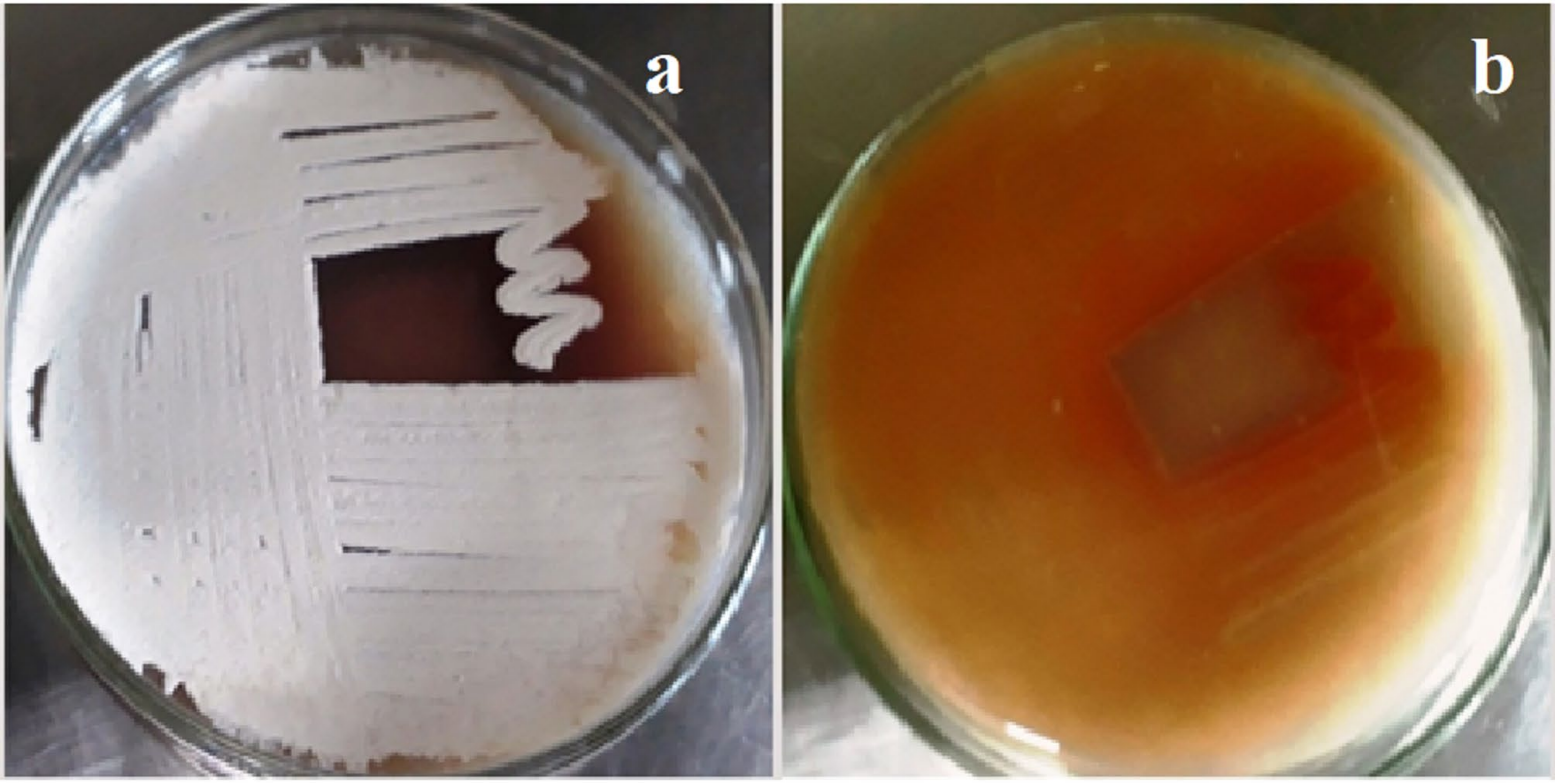
Morphology of *Streptomyces microflavus* strain BA2 colony. Aerial mycelium (**a**) and substrate mycelium (**b**).

The isolate exhibits a powdery appearance, white aerial mycelium, brown substrate mycelium, and the synthesis of melanin pigment (Fig. 1). The substrate mycelium of colonies developed within two days of incubation. By the third to fifth day, the colonies resembled typical bacterial growth. Confirmation of an actinomycetal colony can be achieved by assessing its characteristic powdery texture.

### Sequencing 16 S rRNA genes for molecular identification

#### *Streptomyces microflavus* strain BA2 16S rRNA sequence analysis

**Fig. 2:**
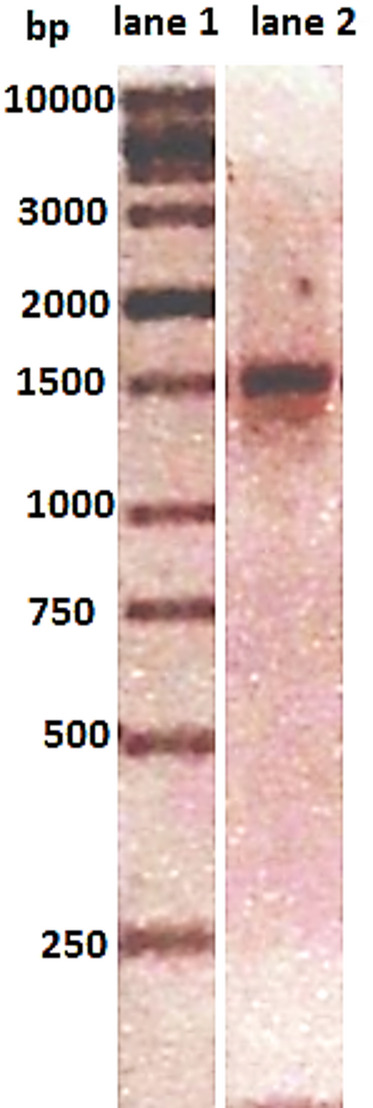
PCR-amplified 16 S rRNA gene. Lane 1: Molecular weight marker (SiZer-1000 DNA marker); Lane 2: Amplified DNA fragment (~ 1500 bp) from a single colony of *S. microflavus* strain BA2.

**Fig. 3:**
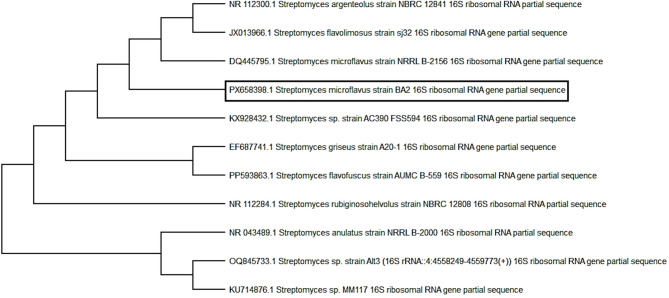
A phylogenetic tree of* S. microflavus *strain BA2 illustrates how close* S. microflavus *strain BA2 against other *Streptomyces *neighbors. It has been reconstructed using MEGA-X software.

A 1500-bp segment of the 16 S rRNA gene was amplified by PCR (Fig. 2). The Foster City, California-based Applied Biosystems 3500xL Genetic Analyzer purified and sequenced amplified DNA. NCBI was used to compare the sequence to other bacterial isolates. DNA sequencing placed the isolate in the Actinobacteria phylum, Streptomycetaceae family, and *Streptomyces* genus, demonstrating a 100% similarity to *S. microflavus* NRRL B-2156, *S. flavolimosus* (99.88%), and *S. argenteolus* (99.75%) in NCBI GenBank. The MUSCLE method matched the sequence with 10 closely related *Streptomyces* species. Kumar et al.^12^ used MEGA-X software to merge their NCBI GenBank sequences for Neighbor-Joining phylogenetic analysis and Kimura 2-parameter evolutionary distance calculations. The phylogenetic tree (Fig. 3) confirmed the isolate’s similarity to *Streptomyces microflavus* strain NRRL B-2156. The partial 16 S rRNA gene sequence of *Streptomyces microflavus* strain BA2 has GenBank code PX658398.

### Antibacterial activity

**Fig. 4:**
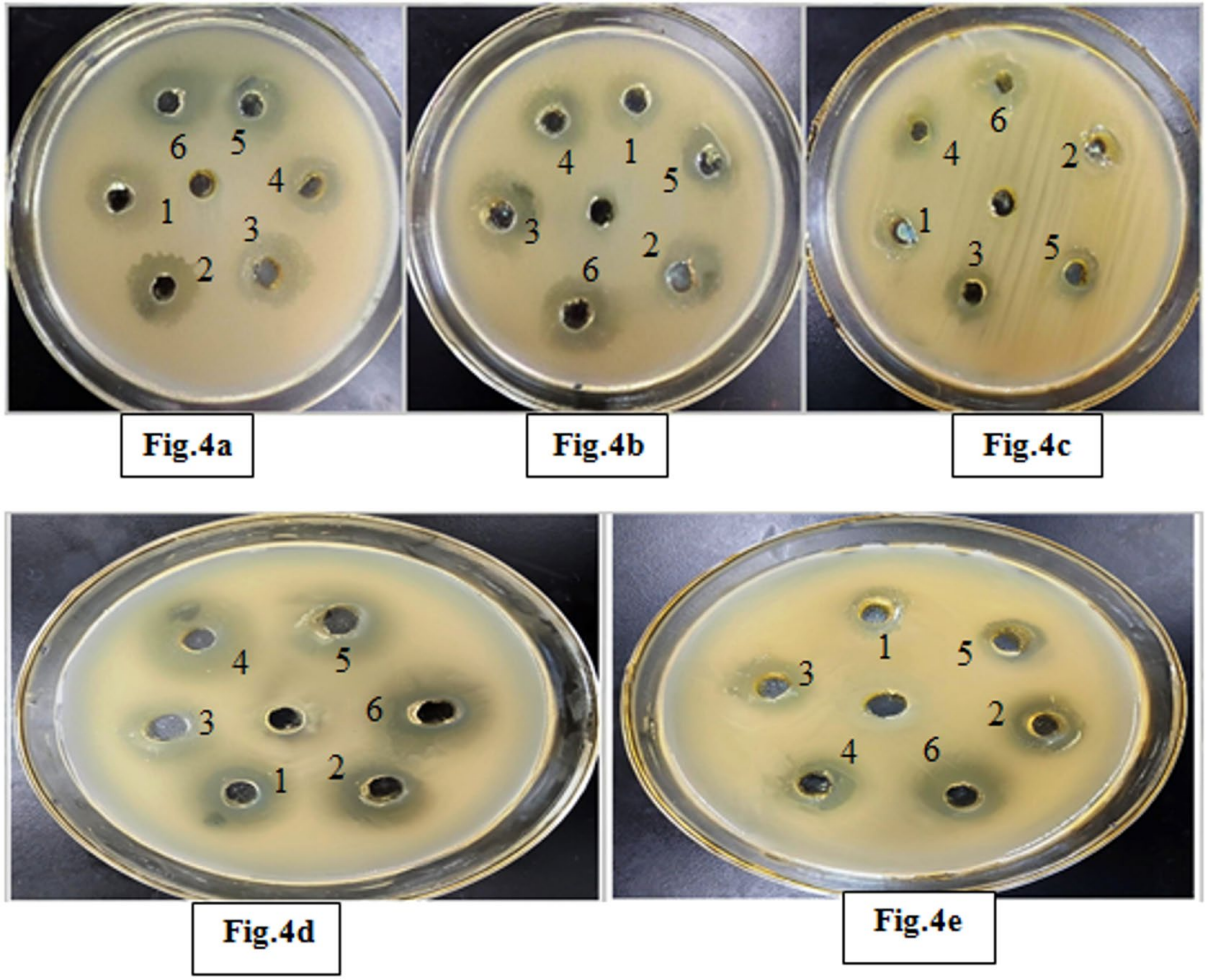
Antibacterial evaluation of aqueous and organic layers of n-butanol, ethyl acetate, and diethyl ether against tested pathogens. Figure 4a: *P. mirabilis*, Fig. 4b: *S. typhi*, Fig. 4c: *E. coli*, Fig. 4d: *S. aureus*, and Fig. 4e: *K. pneumoniae*. 1: n-butanol organic phase, 2: n-butanol aqueous phase, 3: ethyl acetate organic phase, 4: ethyl acetate aqueous phase, 5: diethyl ether organic phase, and 6: diethyl ether aqueous phase.

Table (1) and Figure (4) highlight the potent antibacterial effects of *S. microflavus* BA2 filtrate, with the diethyl ether aqueous fraction emerging as a promising candidate for further exploration. The pathogen-specific responses suggest selective activity, warranting additional studies on compound isolation and potential synergistic formulations. *Streptomyces microflavus* BA2 filtrate exhibited the strongest antibacterial effects across all tested pathogens, with the largest inhibition zones (up to 37 mm for *E. coli*). Among the fractionated extracts, diethyl ether aqueous phase has demonstrated the highest inhibition against multiple pathogens, particularly *P. mirabilis* (21 mm) and *S. aureus* (23 mm). The n-butanol organic phase displayed relatively weak activity across all pathogens (6–11 mm), suggesting that fewer active compounds are extracted into this phase, while the ethyl acetate organic phase showed moderate inhibition, particularly against *S. aureus* (20 mm) and *S. typhi* (17 mm), indicating selective antimicrobial properties. Diethyl ether phases exhibited superior antibacterial activity, with the aqueous fraction generally outperforming the organic fraction.

**Table 1:** Antibacterial evaluation of *S. microflavus* filtrate (mm), aqueous, and organic layers of n-butanol, ethyl acetate, and diethyl ether against *S. aureus*,* K. pneumoniae*,* P. mirabilis*,* S. typhi*, and *E. coli*.

Pathogen	Inhibition zone (mm)							
	S. microflavus filtrate	*N*-butanol organic phase	*N*-butanol aqueous phase	Ethyl acetate organic phase	Ethyl acetate aqueous phase	Diethyl ether organic phase	Diethyl ether aqueous phase	F-value
*S. typhi*	21 ± 0.25^**a**^	7 ± 0.55^**d**^	16 ± 0.33^**b**^	15 ± 0.23^**b**^	17 ± 0.22^**b**^	12 ± 0.38^**c**^	18 ± 0.50^**b**^	42.6
*E. coli*	37 ± 0.33^**a**^	11 ± 0.28^**d**^	8 ± 0.40^**d**^	12 ± 0.29^**c**^	13 ± 0.50^**c**^	11 ± 0.55^**d**^	14 ± 0.34^**c**^	58.4
*P. mirabilis*	19 ± 0.33^**b**^	6 ± 0.50^**d**^	19 ± 0.44^**b**^	14 ± 0.40^**c**^	16 ± 0.51^**c**^	19 ± 0.25^**b**^	21 ± 0.37^**a**^	31.2
*K. pneumoniae*	31 ± 0.30^**a**^	6 ± 0.44^**d**^	8 ± 0.30^**d**^	6 ± 0.39^**d**^	14 ± 0.34^**c**^	7 ± 0.35d	17 ± 0.44^**b**^	27.9
*S. aureus*	25 ± 0.50^**a**^	7 ± 0.30^**d**^	20 ± 0.21^**b**^	19 ± 0.44^**b**^	20 ± 0.29^**b**^	13 ± 0.20^**c**^	23 ± 0.29^**a**^	36.7

*Different letters (a, b, c, d) indicate significant differences between treatments within each strain (Tukey HSD).±Standard errors of means.*

### Determination of minimum inhibitory concentration (MIC)

The aqueous extract has shown a notably good antibacterial activity against the tested pathogens with MIC values of 0.06–0.5 µg/ml, which are more potent than the reference antibiotic ampicillin 0.13–1 µg/ml (Table 2).

**Table 2:** Minimum inhibitory concentration (MIC) of tested samples.

Sample	MIC (µg/ml)				
	*S. typhi*	*E. coli*	*P*. *mirabilis*	*K. pneumoniae*	*S. aureus*
Aqueous extract	0.06	0.25	0.5	0.13	0.13
Ampicillin	0.13	0.5	1	0. 5	0.25

### Antitumor activity

**Fig. 5:**
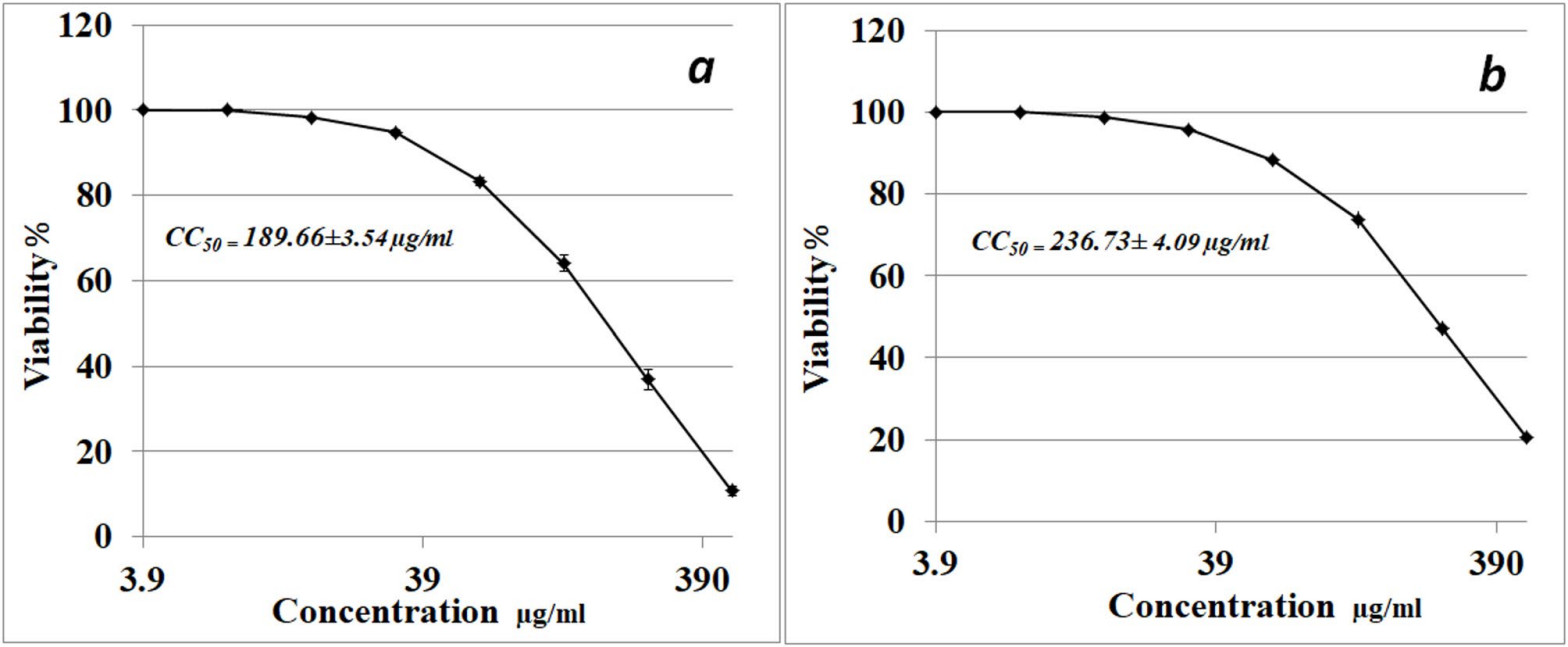
Impact of the di ethyl ether aqueous extract from *Streptomyces microflavus* BA2 on the HepG-2 tumor cell line after a 48-hour incubation period (**a**). Impact of precipitated protein from *Streptomyces microflavus* BA2 on the HepG-2 tumor cell line after a 48-hour incubation period (**b**).

Figure (5) shows the effect of both aqueous extract (a) and precipitated protein (b) of *S. microflavus* BA2 on HepG-2 tumor cell lines. The data obtained show that the aqueous extracts of diethyl ether from *Streptomyces microflavus* BA2 bacteria significantly affect hepatocellular carcinoma cells (HepG-2 cell line) with an IC_50_ value of 189.66 µg/ml, indicating moderate antitumor activity.

Viability drops sharply at higher concentrations, showcasing dose-dependent inhibition. The observed data proved the extract’s cytotoxic potential and establishes its effectiveness against cancer cells. Although the precipitated protein exhibited an IC_50_ value 236.73 µg/ml, it is less potent than the aqueous extract. This reflects its weaker toxic effect on HepG-2 cells.

### Cytotoxic evaluation

**Fig. 6:**
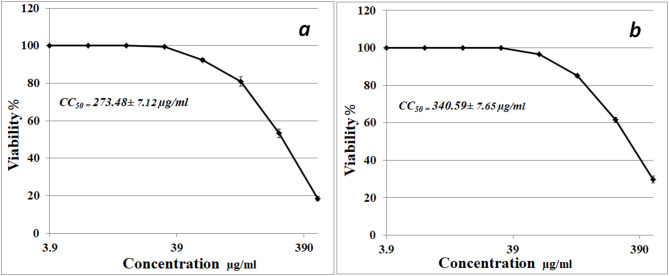
The cytotoxicity impact of diethyl ether aqueous extract and precipitated proteins of *S. microflavus* BA2 on the WI-38 cell line after a 48-hour incubation period. Aqueous solution (**a**) and precipitated protein (**b**).

The effect of aqueous extract (a) and precipitated protein (b) of *S. microflavus* BA2 on WI-38-cell line is shown in Figure (6). Recorded data showed that the cytotoxic effects have a moderate impact with a CC_50_ value of 273.48 µg/ml. The extract displays a safer profile for normal cells. Precipitated protein showed higher CC_50_ 340.59 µg/ml, indicating lower cytotoxicity for normal cells. Viability remains high at lower concentrations, emphasizing a safer profile.

### DPPH radical scavenging activity

**Fig. 7:**
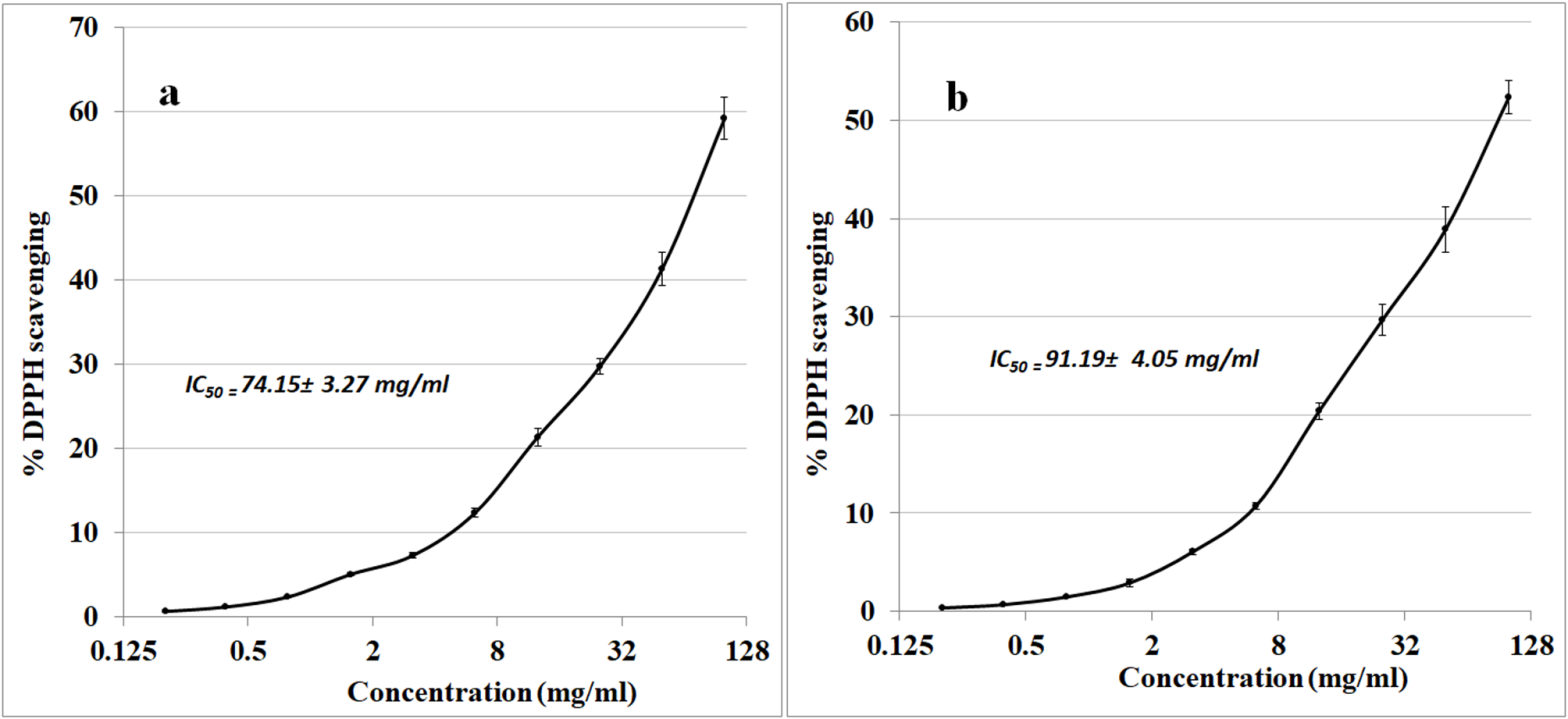
DPPH radical scavenging activity of aqueous extract (**a**) and precipitated protein (**b**) under experimental conditions.

The aqueous extract exhibited DPPH radical scavenging activity with an IC₅₀ value of 74.15 ± 3.27 mg/ml, indicating a moderate antioxidant potential (Fig. 7a). The DPPH radical scavenging activity of precipitated protein has shown a IC₅₀ value of 91.19 ± 4.05 mg/ml, indicating weaker antioxidant potential (Fig. 7b).

### GC-MS analysis

**Fig. 8:**
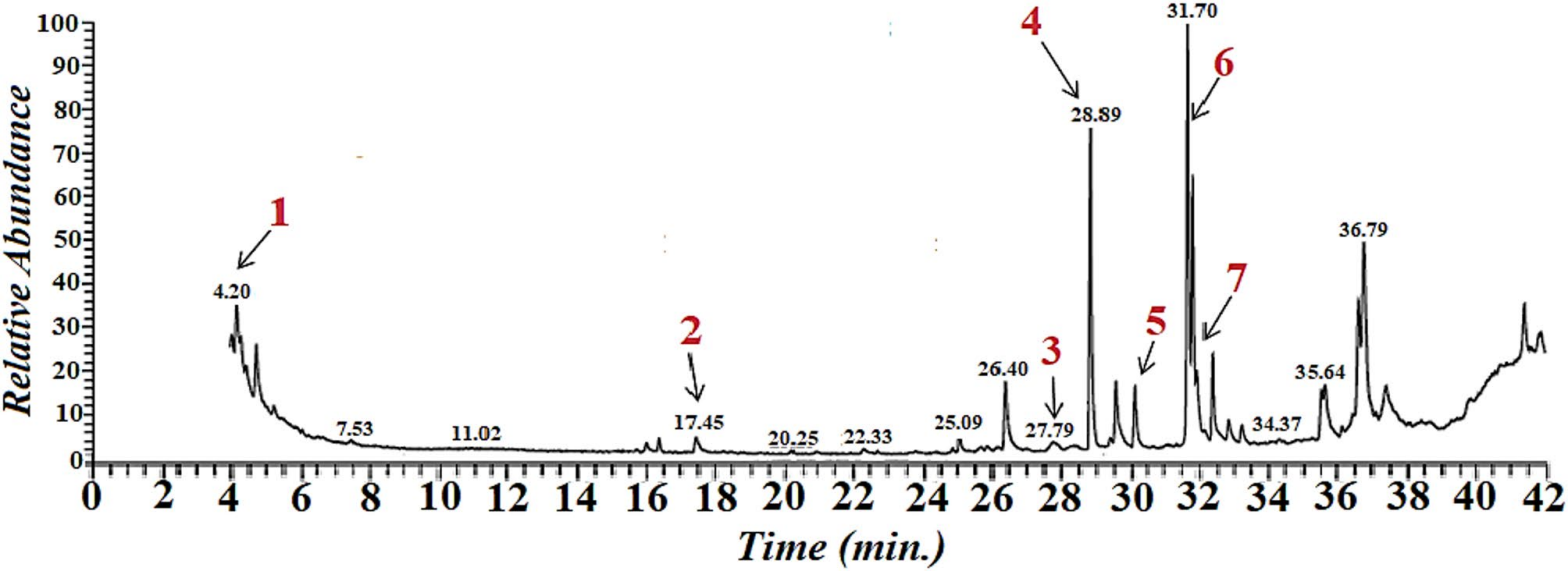
GC-MS chromatography profile of diethyl ether aqueous phase of *Streptomyces microflavus* strain BA2.

The aqueous phase of diethyl ether of *Streptomyces microflavus* BA2 exhibited the most significant antibacterial activity against all tested pathogens. Consequently, it was examined using gas chromatography-mass spectrometry (GC-MS). The acquired GC-MS spectrum of *Streptomyces microflavus* BA2 was juxtaposed with the standard database of the NIST mass spectral library (National Institute of Standards and Technology). The acquired findings validated the presence of seven bioactive chemicals exhibiting antibacterial and antitumor properties (Fig. 8). Table 3 delineates the principal bioactive chemicals found by GC-MS analysis. Hexadecanoic acid methyl ester and (Z)-Oleic acid TMS derivative are the predominant chemicals, with peak areas of 12.68% and 12.19%, respectively. The existence of several chemical classes, such as fatty acids (e.g., eicosapentaenoic acid) and phenolic derivatives (Phenol, 2,4-bis(1,1-dimethylethyl) may explain the extensive range of biological activity. The GC-MS profile (Fig. 8) corroborates the quantitative results from Table 3, validating the existence and relative abundance of bioactive chemicals. The peak area percentages in Table 3 align well with the peak intensities in Figure (8).

**Table 3:** The analysis identified several key bioactive compounds, with the dominant constituents being fatty acids and phenolic derivatives.

*N*	Name of the compound	Molecular formula	Molecular weight	Retention time (Min)	Peak area (%)	Antibacterial and antitumor activities	Sources
1	Benzene, 1-ethyl-3-methyl	C_9_H_12_	120	4.20	2.78	antibacterial	13, 14
2	Phenol,2,4-bis(1,1dimethylethyl)-	C_14_H_22_O	206	17.45	0.78	antibacterial	15, 16
3	Eicosapentaenoic Acid, TBDMS derivative	C_26_H_44_O_2_Si	416	27.85	0.40	both	17, 18
4	Hexadecanoic acid, methyl ester	C_17_H_34_O_2_	270	28.89	12.68	both	19–21
5	Cyclopentaneundecanoic acid, methyl ester	C_17_H_32_O_2_	268	30.15	2.67	antibacterial	22
6	Oleic Acid, (Z)-, TMS derivative	C_21_H_42_O_2_Si	354	31.85	12.19	both	21, 23
7	à-Linolenic acid, TMS derivative	C_21_H_38_O_2_Si	350	31.97	3.04	antibacterial	24

## Discussion

This work identified *Streptomyces microflavus* strain BA2 from the brackish water sediments of Lake Burullus (Egypt), a distinctive habitat with potential for novel microbial discoveries. Morphological and molecular investigations validated its categorization within the phylum Actinobacteria, family Streptomycetaceae, and genus *Streptomyces*, demonstrating a 100% similarity to *S. microflavus* NRRL B-2156, *S. flavolimosus* (99.88%), and *S. argenteolus* (99.75%) in NCBI GenBank. Bergey’s Manual of Systemic Bacteriology^13^ states that *Streptomyces argenteolus* exhibits grey aerial mycelium, a colorless to grayish yellow-green reverse side of the colony, and does not produce melanoid pigments on starch nitrate agar (SNA) media. *Streptomyces flavolimosus* is characterized by the absence of melanin formation on salts-starch agar as well. Conversely, the *Streptomyces microflavus* strain BA2 exhibited white aerial mycelium, brown substrate mycelium, and melanin pigment synthesis on SNA media.

The filtrate of *S. microflavus* has shown antibacterial efficacy against the severe and multidrug-resistant pathogens *S. aureus*,* K. pneumoniae*,* P. mirabilis*,* S. typhi*, and *E. coli.* The aqueous phase of diethyl ether exhibited the most potent inhibitory action among the solvent extracts. *E. coli* exhibited significant sensitivity to the crude extract (37 mm inhibitory zone) but shown reduced sensitivity to the solvent fractions, hence affirming synergistic interactions among the bioactive components. *P. mirabilis* demonstrated extensive sensitivity, indicating the existence of several effective antibacterial agents. The large inhibitory zones seen in the filtrate corroborate the concept that many active components are functioning synergistically^14^. The aqueous extract has shown a notably good antibacterial activity against the tested pathogens with MIC values of 0.06–0.5 µg/ml, which are more potent than the reference antibiotic ampicillin 0.13–1 µg/ml. Fractionation study revealed differing antibacterial effectiveness across several solvents. The diethyl ether extracts presumably included potent hydrophobic chemicals, the ethyl acetate extracts had moderate activity, and the n-butanol fractions demonstrated mild inhibition, indicating reduced quantities or solubility of the bioactive molecules. The aqueous diethyl ether extract of *Streptomyces microflavus* BA2 significantly affects hepatocellular carcinoma cells (HepG-2 cell line) with an IC_50_ value 189.66 µg/ml, confirming moderate antitumor activity. While the precipitated protein shows an IC50 value of 236.73 µg/ml, it is less potent than that of the aqueous extract. The obtained IC₅₀ value (189.66 µg/ml) indicates moderate cytotoxic activity according to commonly accepted cytotoxicity criteria for crude extracts. In comparison, standard chemotherapeutic agents such as Doxorubicin and Cisplatin typically exhibit IC₅₀ values in the low micromolar or even nanomolar range against sensitive cancer cell lines, reflecting substantially higher potency. According to the National Cancer Institute (NCI) guidelines, crude extracts are generally considered promising when IC₅₀ values are below 20–30 µg/ml. Therefore, the observed IC₅₀ of 189.66 µg/ml suggests limited potency in its crude form. However, this level of activity may still indicate the presence of bioactive constituents that could demonstrate enhanced cytotoxic effects after purification, structural optimization, or synergistic combination. Thus, while the cytotoxic effect is moderate, the extract may serve as a preliminary source for further bioassay-guided fractionation and compound isolation. Cytotoxicity is a fundamental aspect of antibacterial research. Numerous effective antibacterial drugs have harmful effects on eukaryotic cells^15^. This study’s cytotoxicity assessment revealed a moderate impact on WI-38 cells, with CC_50_ values 273.48 and 340.59 µg/ml for the aqueous extract and the precipitated protein, respectively. The Selectivity Index (SI = CC₅₀/IC₅₀) was calculated using the obtained CC₅₀ value of 273.48 µg/ml and IC₅₀ value of 189.66 µg/ml, resulting in an SI of 1.44. This value indicates moderate selectivity toward cancer cells. Although the SI does not exceed the commonly suggested threshold of 2 for strong selectivity, the result suggests that the extract exhibits slightly greater toxicity toward cancer cells than normal cells. This result enhances the need for further bioassay-guided fractionation to potentially enhance selectivity and therapeutic safety. Although the extract demonstrated moderate cytotoxic activity, the underlying molecular mechanisms were not experimentally investigated in the present study. The suggested mechanisms, including reactive oxygen species (ROS) generation and apoptosis induction, are hypothetical interpretations based on previously reported studies of bioactive metabolites derived from *Streptomyces* species and other natural products. Several studies have shown that secondary metabolites from *Streptomyces* spp. can induce oxidative stress, disrupt mitochondrial membrane potential, activate caspase cascades, and modulate pro- and anti-apoptotic proteins, ultimately leading to programmed cell death. However, these mechanisms remain speculative in the context of the current work and require experimental validation. Future studies should therefore focus on mechanistic investigations to elucidate the precise pathways involved. Such studies may include intracellular ROS quantification (e.g., DCFH-DA assay), Annexin V/PI flow cytometric analysis for apoptosis detection, assessment of mitochondrial membrane potential, evaluation of caspase-3 and caspase-9 activation, analysis of Bax/Bcl-2 protein expression ratios, and gene expression profiling of apoptosis- and oxidative stress-related markers (e.g., p53, BAX, BCL2, and CASP3). These investigations would provide deeper insight into the molecular basis of the observed cytotoxicity and further clarify the therapeutic potential of the extract. The aqueous extract exhibited DPPH radical scavenging activity with an IC₅₀ value of 74.15 ± 3.27 mg/ml, indicating a moderate antioxidant potential. The precipitated protein has shown an IC₅₀ value of 91.19 ± 4.05 mg/ml, indicating weaker antioxidant potential. Therefore, future investigations incorporating multiple complementary assays such as ABTS, FRAP, and ORAC, along with appropriate reference standards, are necessary to provide a more comprehensive assessment of antioxidant potential.

Many bioactive secondary metabolites, such as polyphenols/phenolic compounds, flavonoids, vitamins, polysaccharides, and peptides, are thought to be responsible for the antioxidant capacities of bacterial extracts^16^. These substances reduce oxidative damage by disrupting the chain reactions of free radicals. The total antioxidant potential of the bacterial extract is determined by the particular makeup of these components^17^. Gas chromatography and mass spectrometry revealed notable antimicrobial and antitumor fatty acids, such as eicosapentaenoic acid (0.40%), hexadecanoic acid methyl ester (12.68%), cyclopentanodecanoic acid methyl ester (2.67%), oleic acid (12.19%), and alpha-linolenic acid (3.04%). Aromatic and phenolic bioactive chemicals, including benzene (1-ethyl-3-methyl, 2.78%) and phenol (2,4-di(1,1-dimethylethyl), 0.78%) were identified, underscoring the variety of antimicrobial and antitumor metabolites in the original extracts. The effectiveness of these chemicals aligns with prior research on *Streptomyces* species. Prole et al.^18^ investigated four novel *Streptomyces* strains derived from Masher pasture soil. The ethyl acetate extracts demonstrated significant efficacy against several pathogens, underscoring the value of solvent fractionation. Kalyani et al.^19^ and Rammali et al.^20^ identified a similar scenario, investigating the antibacterial efficacy of *Streptomyces* isolates from saline soil, which had a significant inhibitory impact on resistant bacterial strains.

Eicosapentaenoic acid (EPA), an omega-3 polyunsaturated fatty acid, has antibacterial properties against *Bacillus subtilis*,* Listeria monocytogenes*,* Staphylococcus aureus*, and *Pseudomonas aeruginosa*, with minimum inhibitory concentration (MIC) values between 500 and 1350 µg/ml^21^. Lauritano et al.^22^ proved that EPA was produced by *Myctophum punctatum*, with a high anticancer activity against Lung cell line A549 and breast cell line MCF7. Hexadecanoic acid methyl ester, identified in ethanolic extracts of *P. maritimum* seeds, exhibited minimum inhibitory concentrations ranging from 25 to over 50 µg/ml against *E. coli* and *C. krusei*^23^. A similar situation was noted in the study by Atallah et al.^24^. This chemical was isolated from ethyl acetate extracts of *Streptomyces griseorubens* derived from Egyptian black sand and shown antibacterial properties against *B. subtilis*,* Salmonella enteritidis*, and *P. aeruginosa.* Cyclopentanondecanoic acid methyl ester in *Monascus purpureus* extracts exhibited antibacterial activity against *S. typhimurium* (MIC/MBC: 6.25/12.5 µg/ml)^25^. Oleic acid has inhibitory effects on *Bacillus cereus*,* Staphylococcus aureus*,* Escherichia coli*,* Salmonella typhi*,* Candida albicans*, and *Aspergillus flavus*^26^. Linolenic acid, present in heat-treated and fermented soybeans with *Rhizopus oligosporus*, suppresses the proliferation of *S. aureus* and *B. subtilis*^27^. Nisa et al.^28^ found that hexadecanoic acid ethyl ester and oleic acid that were detected in the crude extract of *Arisaema flavum* exhibited a strong anticancer activity against human breast cancer cell line MCF-7.

Traditional antibacterial techniques include impeding bacterial growth by obstructing cell wall production, protein synthesis, and/or diverse metabolic processes inside the cell. Furthermore, unconventional mechanisms such as the suppression of horizontal gene transfer (HGT), quorum sensing (QS), and efflux pump activity may play a role in diminishing bacterial resistance^29^. Benzene, 1-ethyl-3-methyl, an aromatic hydrocarbon, was detected in ethyl acetate extracts of *Streptomyces* species from mangrove sediments in the Egyptian Red Sea and exhibited antimicrobial efficacy against *Vibrio damselflies*,* Staphylococcus aureus*,* Enterococcus faecalis* and *Candida albicans* (MIC: 285–545 µg/ml)^30^. Yang et al.^31^ indicated that aromatic chemicals engage with bacterial membranes via hydrophobic interactions, hence augmenting their antibacterial efficacy. The compound 2,6-di(1,1-dimethyl ethyl) phenol, identified in *Pseudomonas fluorescens* TL-1, has significant antifungal efficacy against plant infections^32^. The presence of *Cotzeneria* bacteria, supported by computer docking studies, has confirmed their role in inhibiting mitochondrial ATP synthase, thereby enhancing antibacterial activity^33^.

According to Al-shaibani^34^, the mechanism of action of anticancer compounds produced by *Streptomyces* spp. included the formation of reactive oxygen species (ROS), activation of tumor suppressor proteins like p53 and p21, inhibition of topoisomerase II (TOP II) and DNA intercalation, which prevent normal replication and transcription, inhibition of RNA polymerase, and activation of caspases, which induce apoptosis.

Future studies involving bioassay-guided fractionation, purification, and comprehensive spectroscopic characterization are warranted to confirm the structures of different bioactive compounds produced by *Streptomyces microflavus* strain BA2 and determine their individual biological contributions.

## Materials and methods

### Samples collection and isolation

Burullus Lake is the second biggest lake on the northern side of the Nile Delta in Egypt. Samples were crushed and sieved through 2 mm holes after 34 h of air-drying at 35°C. Soil particles were used for isolation, and their sizes varied between 0.1 and 2 μm. According to Williams et al.^35^, one gram of sieved soil particles was suspended in nine milliliters of sterile distilled water. We ran serial dilutions all the way up to 10^− 4^ dilutions. According to Williams and Davies^36^, 50 µl of each dilution was put over starch nitrate agar, and then it was left to incubate at 30 °C for seven days. Colonies were isolated and analyzed.

### Morphological characterization

Characteristics like growth rate, pigmentation, texture, substrate mycelium, and aerial mycelium were studied on starch-nitrate medium to determine the morphological features. The colors of the aerial and reverse cultures were also studied under normal illumination conditions, as pointed out by Shiriling and Gottlieb^37^.

### Molecular detection

Centrifugation at 3000 g for 20 min was used to assemble *Streptomyces microflavus* strainBA2, which had been cultivated on starch-nitrate broth medium for seven days. Before genomic DNA could be extracted using EZ-10 Spin Column Bacterial Genomic DNA Miniprep Kits, the resulting pellet was washed twice with sterile deionized water. The universal forward primer 27 F (5’-AGAGTTTGATC (AC) TGGCTCAG–3’) and the reverse primer 1492R (5’-ACGG (CT) TACCTTGTTACGACTT-3’) were used to amplify the 16 S rRNA regions according to the method outlined by Al-Dhabi et al.^38^.

### Antibacterial evaluation of *Streptomyces microflavus *strain BA2 

The Microbial Biotechnology Unit of the Faculty of Science, Kafrelsheikh University, generously provided five multi-drug-resistant pathogens: *Salmonella typhi*,* Escherichia coli*,* Proteus mirabilis*,* Klebsiella pneumoniae*, and *Staphylococcus aureus*. Within a 250 ml Erlenmeyer flask, 50 ml of autoclaved starch-nitrate broth medium was used to cultivate 1 ml of *Streptomyces microflavus* strain BA2 spore suspension. For seven days, the culture was vibrated at 30 °C and 120 rpm. Centrifugation at 3000 g for 20 min separated the biomass from the supernatant after the incubation period. For the purpose of testing bacterial pathogens, 1 ml of broth cultures that were 18 h old was swabbed individually onto newly produced nutritional agar medium. A sterile cork drill was used to drill 5 mm wells in independently infected plates.The filtrate was added to each well at a volume of 100 µl. For 24 h, the plates were placed in an incubator set at 37 °C. The diameter of the inhibitory zone was measured in millimeters after the incubation time^39^.

### Partial purification of antibacterial compounds

An inoculum of one liter of culture broth was prepared by growing *Streptomyces microflavus* strain BA2, which has already undergone partial purification of its antibacterial chemicals. After that, the culture was kept at 30 °C and 120 rpm in a rotary shaker incubator for a duration of 7 days. After separating the filtrate from the biomass of *Streptomyces microflavus* strain BA2, the mixture was centrifuged at 8000 g for 15 min at 4 °C. The filter paper used was Whatman no. 1. After aseptically transferring the supernatant to 250 ml flasks, the filtrate was treated with three distinct solvents: n-butanol, ethyl acetate, and diethyl ether, each supplied in equal amounts (v/v). Phase separation was achieved by aggressively shaking the filtrate and solvent combination for 20 min and then keeping it stationary for an additional 15 min. To create a gummy crude extract, the organic and watery components were evaporated in a 40 °C oven until the solvent was removed. After the leftover residues were dried in a vacuum desiccator, they were weighed. A tiny amount of methanol (1 mg/ml) was then added to redisperse them, and the well diffusion technique was used to assess their antibacterial activity. Every test included the use of methanol as a control for the pathogenic microorganisms that were examined. In order to extract antibacterial chemicals, the optimal solvent was chosen and applied^40^. Using a rotary evaporator (Heidolph, Germany), the organic and water phases were combined and concentrated by evaporation until they were almost dry at decreased pressure.

### Determination of minimum inhibitory concentration (MIC)

The CLSI-described standard broth micro-dilution method was used to estimate the MIC values against all tested pathogens. In short, the disc diffusion test was used to create the bacterial suspensions, which were subsequently diluted 1:1000 to yield 1^–2^ × 105 CFU ml^− 1^. The minimum inhibitory concentration (MIC) values ​​for the studied extract were calculated using the binary serial dilution method at concentrations ranging from 0.0625 to 64 µg/ml per sample. The tested bacteria were cultivated on nutrient broth in a shaking incubator set at 150 rpm. For 20 h, the cultures were incubated at 37 °C. Optical density (OD) measurements at 600 nm were used to estimate the results^41^. Ampicillin was used as a reference compound.

### Total protein extraction

The cell-free culture supernatant was treated with ammonium sulfate at a concentration of 40% to get total proteins. According to Ahmad et al.^42^, the protein pellets were retrieved by centrifugation at 14,000 g for 30 min at 4 °C. Subsequently, they were dissolved in 5 ml of distilled water and kept in a refrigerator at 4 °C.

### Antitumor activity of aqueous extract and total proteins *in vitro*

Hepatocellular carcinoma cells (HepG-2 tumor cell lines) were obtained from the American Type Culture Collection (ATCC, Rockville, MD). The cells were maintained at 37 °C in a humidified atmosphere containing 5% carbon dioxide and were re-cultured two to three times weekly. Tumor resistance testing was performed according to the Mosmann protocol^43^.

### Cytotoxic effect assessment

The cytotoxicity of both aqueous extract and total proteins were examined in human lung fibroblast normal cells (WI-38 cell line) using the protocols established by Gomha et al.^44^.

### Antioxidant activity

The DPPH radical scavenging activity of the aqueous extract and total proteins was determined using the method of Al Zahrani et al.^45^.

### Analyzing the aqueous extract using GC-MS

The National Institute of Oceanography and Fisheries in Alexandria, Egypt, used GC-MS to discover bioactive compounds from *Streptomyces microflavus* strain BA2 using the method of Hassan and Shobier^46^.

### Evaluation using statistical methods

All experiments were performed in triplicate (*n* = 3 independent biological replicates), and each measurement was conducted in three technical replicates where applicable. Data are presented as mean ± standard errors^47^. Statistical analysis was carried out using SPSS software, which stands for Statistical Package for the Social Sciences. Differences between groups were evaluated using one**-**way analysis of variance (ANOVA) followed by Tukey’s post hoc test. A value of p *<* 0.05 was considered statistically significant.

## Conclusion

In this study, *Streptomyces microflavus* strain BA2 wasn’t proposed as a novel species, but rather as a biologically and functionally distinct strain within *S. microflavus*, with unique metabolomic and pharmacological characteristics. BA2 was isolated from brackish sediment of Lake Burullus (Egypt), an environment that differs substantially from the habitats of previously described strains. GC–MS analysis revealed the presence of seven bioactive compounds, including multiple fatty acids and phenolic derivatives associated with antibacterial and anticancer activities. The current strain, *Streptomyces microflavus* strain BA2, demonstrated broad-spectrum antibacterial activity against both Gram-positive and Gram-negative pathogens, moderate antioxidant activity, and selective cytotoxicity against HepG-2 liver cancer cells with comparatively lower toxicity toward normal WI-38 fibroblasts. This multifunctional bioactivity profile distinguishes BA2 at the strain level. These findings underscore the novelty of brackish-water-derived *Streptomyces* as a sustainable source of dual antimicrobial and anticancer agents capable of addressing antibiotic resistance and cancer therapeutics. Overall, this work provides an initial functional characterization of strain BA2 and highlights its potential as a source of bioactive metabolites. Future studies involving bioassay-guided fractionation, structural elucidation of purified compounds, multi-cell-line evaluation, mechanistic analyses, and in vivo safety assessment will be essential to determine its true therapeutic relevance.

## Supplementary Information

Below is the link to the electronic supplementary material.

Supplementary Material 1

## Data Availability

Data was provided within the manuscript or supplementary information files.Sequence data that supports the findings of this study have been deposited in the NCBI’s Sequence Read Archive under accession number PX658398.

## References

[1] Murray, C. J. L. et al. Global burden of bacterial antimicrobial resistance in 2019: A systematic analysis. *Lancet* **399**, 629–655 (2022). 35065702 10.1016/S0140-6736(21)02724-0 PMC8841637

[2] Rodrigues, R. S. et al. A. D. L. Biotechnological potential of actinomycetes in the 21st century: A brief review. *Antonie van Leeuwenhoek*. **117**, 82 (2024). 38789815 10.1007/s10482-024-01964-y

[3] Selim, M. S. M., Abdelhamid, S. A. & Mohamed, S. S. Secondary metabolites and biodiversity of actinomycetes. *J. Genet. Eng. Biotechnol.* **19**, 72 (2021). 33982192 10.1186/s43141-021-00156-9 PMC8116480

[4] Jagannathan, S. V., Manemann, E. M., Rowe, S. E., Callender, M. C. & Soto, W. Marine actinomycetes: New sources of biotechnological products. *Mar. Drugs* **19**, 365 (2021). 34201951 10.3390/md19070365 PMC8304352

[5] Pan, C., Hassan, S. S. U., Ishaq, M., Yan, S. & Jin, H. Marine actinomycetes: A hidden treasure trove for antibacterial discovery. *Front. Mar. Sci.* **12**, 1558320 (2025).

[6] Diab, M. K., Mead, H. M., Khedr, M. M. A., Abu-Elsaoud, A. M. & El-Shatoury, S. A. Actinomycetes as a natural resource for sustainable pest control and safeguarding agriculture. *Arch. Microbiol.* **206**, 268 (2024). 38762847 10.1007/s00203-024-03975-9

[7] Girma, D. et al. Antibacterial and antioxidant applications of pigment-producing actinomycetes from cave sediments. *BMC Microbiol.* **25**, 236 (2025). 40269685 10.1186/s12866-025-03959-9 PMC12016302

[8] Atallah, B., Haroun, S. & El-Mohsnawy, E. Antibacterial activity of two actinomycetes species isolated from black sand in North Egypt. *S. Afr. J. Sci.* **119**, 11–12 (2023).

[9] El-Mohsnawy, E. et al. Assignment of the antibacterial potential of Ag_2_O/ZnO nanocomposite against MDR bacteria Proteus mirabilis and Salmonella typhi isolated from bone marrow transplant patients. *Braz. J. Microbiol.* **54**, 2807–2815 (2023). 37801221 10.1007/s42770-023-01138-4 PMC10689719

[10] Helmi, N. R. Exploring the diversity and antimicrobial potential of actinomycetes from Saudi Arabia. *Front. Microbiol.* **16**, 1568899 (2025). 40207161 10.3389/fmicb.2025.1568899 PMC11979186

[11] Ibrahim, A. M., El-Sayed, M. A. & El-Gendy, A. O. Advances in isolation and screening of marine actinomycetes for drug discovery. *Microb. Biotechnol.* **18**, 455–468 (2025).

[12] Kumar, S., Stecher, G. & Tamura, K. MEGA7: Molecular evolutionary genetics analysis version 7.0 for bigger datasets. *Mol. Biol. Evol.* **33**, 1870–1874 (2016). 27004904 10.1093/molbev/msw054 PMC8210823

[13] Bergey’s Manual of Systematic Bacteriology. *The Actinobacteria* Vol. 5 (Springer, 2012).

[14] Hassan, M. G. et al. Antimicrobial, antibiofilm, cytotoxicity, and anti-DNA topoisomerase activity of *Streptomyces* sp. 22SH with ADME and in silico study. *BMC Microbiol.* **25**, 219 (2025). 40240972 10.1186/s12866-025-03912-w PMC12001559

[15] Zipperer, A. & Kretschmer, D. Cytotoxicity assays as predictors of the safety and efficacy of antimicrobial agents. *Methods Mol. Biol.* **1520**, 107–118 (2017). 27873248 10.1007/978-1-4939-6634-9_6

[16] Dat, T. T. H. et al. Pharmacological properties, volatile organic compounds, and genome sequences of bacterial endophytes from the mangrove plant Rhizophora *apiculata Blume*. *Antibiotics* **10**, 1491 (2021). 34943703 10.3390/antibiotics10121491 PMC8698355

[17] Gulcin, I. & Alwasel, S. H. DPPH radical scavenging assay. *Processes* 2248 (2023).

[18] Prole, J. R., Allenby, N., Manning, D. A. & Goodfellow, M. Identification of four novel *Streptomyces* isolated from machair grassland soil using a culture-based bioprospecting strategy: *Streptomyces caledonius* sp. nov., *Streptomyces machairae* sp. nov., *Streptomyces pratisoli* sp. nov. and *Streptomyces achmelvichensis* sp. nov.. *Int. J. Syst. Evol. Microbiol.* 4 (2025). 10.1099/ijsem.0.006736 PMC12281783 40208665

[19] Kalyani, B. S., Krishna, P. S., Laxminarayana, E. & Sreenivasulu, K. Novel bioactive compounds from *Streptomyces* sp. NLKPB45 isolated from marine soil sediment. *Pharm. Chem. J.* **57**, 834–841 (2023).

[20] Rammali, S. et al. Exploring the antimicrobial and antioxidant activities of *Streptomyces* sp. EIZ2 isolated from Moroccan agricultural soil. *Microbiol. Res.* **15**, 762–786 (2024).

[21] Shin, S. Y., Bajpai, V. K., Kim, H. R. & Kang, S. C. Antibacterial activity of bioconverted eicosapentaenoic (EPA) and docosahexaenoic acid (DHA) against foodborne pathogenic bacteria. *Int. J. Food Microbiol.* **113**, 233–6 (2007). 16860896 10.1016/j.ijfoodmicro.2006.05.020

[22] Lauritano, C. et al. First evidence of anticancer and antimicrobial activity in Mediterranean mesopelagic species. *Sci. Rep.* **10**, 4929 (2020). 32188923 10.1038/s41598-020-61515-z PMC7080843

[23] Ertuğrul, M. et al. Antioxidant, antimicrobial, anticancer, and molecular docking insights into *Pancratium maritimum* seeds and flowers: A phytochemical approach. *Chem. Open* **14**, 1–19 (2025). 10.1002/open.202400407 PMC11808260 39790022

[24] Atallah, B., El-Mohsnawy, E., El-shouny, W. & Haroun, S. Identification and characterization of different potentially antibacterial compounds from a marine *Streptomyces* sp. Sp1. *J. Anim. Plant Sci.* **33**, 166–173 (2023).

[25] Sehim, A. E. et al. GC-MS analysis, antibacterial, and anticancer activities of *Hibiscus sabdariffa* L. methanolic extract: In vitro and in silico studies. *Microorganisms* **11**, 1601 (2023). 37375103 10.3390/microorganisms11061601 PMC10302641

[26] Qanash, H. et al. Effectiveness of oil-based nanoemulsions with molecular docking of its antimicrobial potential. *BioResources* **18**, 1554–1576 (2023).

[27] Kusumah, D. et al. Linoleic acid, α-linolenic acid, and monolinolenins as antibacterial substances in the heat-processed soybean fermented with *Rhizopus oligosporus*. *Biosci. Biotechnol. Biochem.* **84**, 1285–1290 (2020). 32089087 10.1080/09168451.2020.1731299

[28] Nisa, S. et al. Isolation, characterization and anticancer activity of two bioactive compounds from *Arisaema flavum* (Forssk.) Schott. *Molecules* **27**, 7932 (2022). 36432033 10.3390/molecules27227932 PMC9697112

[29] Casillas-Vargas, G. et al. Antibacterial fatty acids: An update of possible mechanisms of action and implications in the development of the next-generation of antibacterial agents. *Prog. Lipid Res.* **2**, 101093 (2021). 10.1016/j.plipres.2021.101093 PMC8137538 33577909

[30] Fahmy, M. N. Isolation and characterization of *Streptomyces* sp. NMF76 with potential antimicrobial activity from mangrove sediment, Red Sea, Egypt. *Egypt. J. Aquat. Biol. Fish.* **24**, 479–495 (2020).

[31] Yang, J., Chen, H., Huang, C., Chen, C. & Chen, Y. Antibacterial activities of functional groups on the benzene rings in nucleic acid nanocarriers. *Mater. Today Chem.* **38**, 102106 (2024).

[32] Ren, J., Wang, J., Karthikeyan, S., Liu, H. & Cai, J. Natural anti-phytopathogenic fungi compound phenol,2,4-bis(1,1-dimethylethyl) from *Pseudomonas fluorescens* TL-1. *Indian J. Biochem. Biophys.* **56**, 162–168 (2019).

[33] Devi, T. S. et al. Antifungal activity and molecular docking of phenol, 2,4-bis(1,1-dimethylethyl) produced by plant growth-promoting *actinobacterium Kutzneria* sp. strain TSII from mangrove sediments. *Arch. Microbiol.* **203**, 4051–4064 (2021). 34046705 10.1007/s00203-021-02397-1

[34] Al-Shaibani, M. M. et al. Anticancer compounds from *Streptomyces*: Insights from metagenomics and mechanistic perspective. *Folia Microbiol (Praha)* **70**, 1159–1172 (2025). 41039183 10.1007/s12223-025-01332-x PMC12769640

[35] Williams, S. et al. Numerical classification of *Streptomyces* and related genera. *Microbiology* **129**, 1743–1813 (1983). 10.1099/00221287-129-6-1743 6631406

[36] Williams, S. T. & Davies, F. L. Use of antibiotics for selective isolation and enumeration of actinomycetes in soil. *J. Gen. Microbiol.* **38**, 251–262 (1965). 14287203 10.1099/00221287-38-2-251

[37] Shirling, E. B. & Gottlieb, D. Methods for characterization of *Streptomyces* species. *Int. J. Syst. Bacteriol.* **6**, 313–340 (1966).

[38] Al-Dhdabi, N., Esmail, G., Duraipandiyan, V., Valan Arasu, M. & Salem-Bekhit, M. Isolation, identification and screening of antimicrobial thermophilic *Streptomyces* sp. Al-Dhabi-1 isolated from Tharban hot spring, Saudi Arabia. *Extremophiles* **20**, 79–90 (2016). 26515082 10.1007/s00792-015-0799-1

[39] Sathiyanarayanan, G., Gandhmathi, R., Sabarathnan, B., SeghalKiran, G. & Selvin, J. Optimization and production of pyrrolidon antimicrobial agent from marine sponge-associated *Streptomyces* sp. MAPSIS. *Bioprocess Biosyst. Eng.* **37**, 561–573 (2014). 23917410 10.1007/s00449-013-1023-2

[40] Holmalahti, J., Von Wright, A. & Raatikainen, O. Variations in the spectra of biological activities of actinomycetes isolated from different soils. *Lett. Appl. Microbiol.* **18**, 144–146 (1994).

[41] CLSI. *Zone diameter Interpretive Standards and corresponding minimal inhibitory concentration interpretive break point* (Clinical and Laboratory Standard Institute) (2012).

[42] Ahmad, M. S. et al. Exploring the antimicrobial and antitumor potentials of *Streptomyces* sp. AGM12-1 isolated from Egyptian soil. *Front. Microbiol.* **8**, 438 (2017). 28348553 10.3389/fmicb.2017.00438 PMC5346535

[43] Mosmann, T. Rapid colorimetric assay for cellular growth and survival: Application to proliferation and cytotoxicity assays. *Immunol. Methods* **65**, 55–63 (1983). 10.1016/0022-1759(83)90303-4 6606682

[44] Gomha, S. M., Riyadh, S. M., Mahmmoud, E. A. & Elaasser, M. M. Synthesis and anticancer activities of thiazoles, 1,3-thiazines, and thiazolidine using chitosan-grafted-Poly(vinylpyridine) as basic catalyst. *Heterocycles* **91**, 1227–1243 (2015).

[45] Al Zahrani, N. A., El-Shishtawy, R. M., Elaasser, M. M. & Asiri, A. M. Synthesis of novel chalcone-based phenothiazine derivatives as antioxidant and anticancer agents. *Molecules* **25**, 4566 (2020). 33036301 10.3390/molecules25194566 PMC7583060

[46] Hassan, S. & Shobier, A. GC/MS identification and applications of bioactive seaweed extracts from Mediterranean coast of Egypt. *EJABF* **5**, 1–21 (2018).

[47] Lorowitz, W., Saxton, E., Sondossi, M. & Nakaoka, K. Integrating statistics with a microbiology laboratory activity. *Microbiol. Educ.* **6**, 14–19 (2005). 23653559 10.1128/me.6.1.14-19.2005 PMC3633138

