# Supplementary material for: Antibacterial and anticancer properties of Streptomyces microflavus BA2 isolated from brackish waters
Source: Sci Rep. 2026 May 20;16:15737. doi: 10.1038/s41598-026-51609-5 (PMC13190683; doi:10.1038/s41598-026-51609-5)
Supplement: Supplementary file 1 — Supplementary Material 1 [file 41598_2026_51609_MOESM1_ESM.docx]

**Antibiotic resistance profile of MDR bacteria used in the study**

| **Isolate** | **Ofloxacin (5 µg)** | **Nitrofurantoin (300 µg)** | **Imipenem (10 µg)** | **Amikacin (30 µg)** | **Vancomycin (30 µg)** | **Tobramycin (10 µg)** | **Norfloxacin (10 µg)** | **Meropenem (10 µg)** | **Ceftazidime (30 µg)** | **Colistin (10 µg)** | **Ceftriaxone (30 µg)** |
| --- | --- | --- | --- | --- | --- | --- | --- | --- | --- | --- | --- |
| ***E. coli*** | 0.0 ± 0.0 | 12 ± 0.9 | 0.0± 0.0 | 14 ±. 0.2 | 0 ± 0 | 0.0 ± 0.0 | 0.0 ± 0.0 | 23 ± 1.1 | 0.0 ± 0.0 | 18 ± 0.6 | 10 ± 0.3 |
| ***K. pneumoniae*** | 0.0 ± 0.0 | 0.0± 0.0 | 0.0 ± 0.0 | 0.0 ± 0.0 | 0 ± 0 | 0.0 ± 0.0 | 0.0± 0.0 | 0.0 ± 0.0 | 15 ±1.3 | 0.0 ± 0.0 | 0.0 ± 0.0 |
| ***S. typhi*** | 0.0 ± 0.0 | 0.0± 0.0 | 0.0 ± 0.0 | 0.0 ± 0.0 | 0 ± 0 | 0.0 ± 0.0 | 0.0± 0.0 | 20± 0.9 | 9± 0.4 | 0.0 ± 0.0 | 0.0 ± 0.0 |
| ***P. mirabilis*** | 12 ± 0.5 | 0.0± 0.0 | 0.0 ± 0.0 | 12 ± 0.7 | 0 ± 0 | 0.0 ± 0.0 | 0.0± 0.0 | 21 ± 1.2 | 7± 0.9 | 16 ± 1.0 | 22 ± 0.8 |
| ***S. aureus*** | 10 ± 0.2 | 0.0± 0.0 | 0.0 ± 0.0 | 0.0 ± 0.0 | 0 ± 0 | 0.0 ± 0.0 | 0.0± 0.0 | 18 ± 0.5 | 10± 0.4 | 0.0 ± 0.0 | 0.0 ± 0.0 |

**Table 1. Inhibition zone diameters (in mm) for each bacterial isolate against the tested antibiotics. Values represent the mean diameter of the clear zone around antibiotic disks. Zones ≤ 14 mm were considered resistant, 15–19 mm intermediate, and ≥ 20 mm sensitive according to CLSI guidelines.**

**
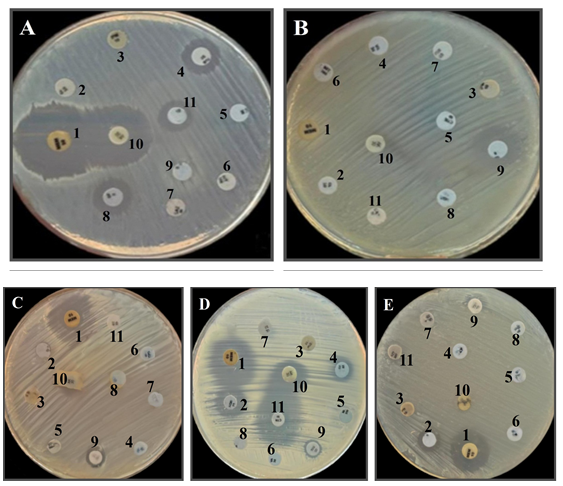
**

**Figure 1. Antibiotic sensitivity test of *E. coli* (A), *K. pneumoniae* (B), *S. typhi* (C), *P. mirabilis* (D), *and S. aureus* (E). MEM (1), OFL** (**2), IPM (3), AK (4), VAN (5), TOB (6), NOR (7), NIT (8), CAZ (9), COL (10), and CRO (11).**

**Serial numbering system**

A total of 20 clinical isolates were obtained from stool of patients suffering diarrhea. Each clinical isolate was given a code with two digits: CI-01 to CI-20 and designated as CI-01 to CI-20 for identification and analysis. Based on biochemical identification, isolate CI-11 was identified as *Escherichia coli*, CI-14 was identified as *Salmonella typhi*, CI-16 was identified as *Proteus mirabilis*, CI-17 was identified as *Klebsiella pneumonia*, and CI-20 was identified as *Staphylococcus aureus*.

**Table 2. Serial numbering system of clinical isolates involved in the study.**

| **Code** | **Clinical source** | **Biochemical identification** |
| --- | --- | --- |
| **CI-11** | Stool | *Escherichia coli* |
| **CI-14** | Stool | *Salmonella typhi* |
| **CI-16** | Stool | *Proteus mirabilis* |
| **CI-17** | Stool | *Klebsiella pneumoniae* |
| **CI-20** | Stool | *Staphylococcus aureus* |

**Original gel picture**


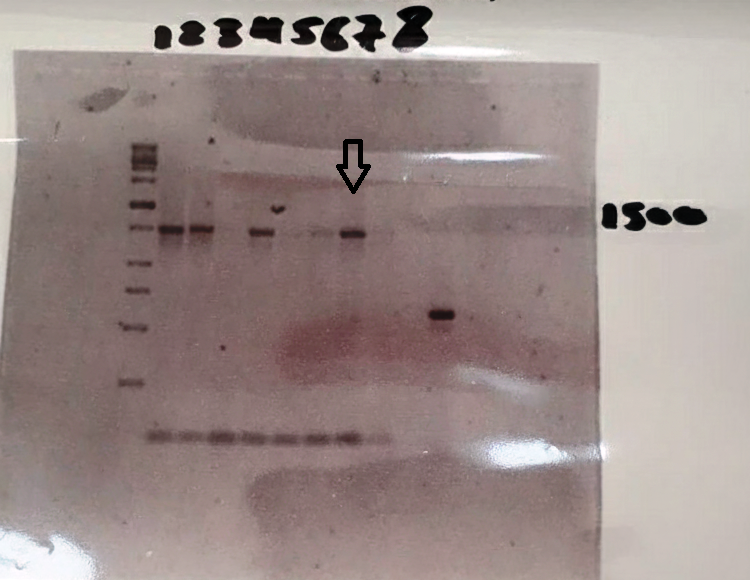


**Figure 2. PCR-amplified 16S rRNA gene. Lane 1: Molecular weight marker (SiZer-1000 DNA marker); Lane 8: Amplified DNA fragment (~1500 bp) from a single colony of *S. microflavus* strain BA2.**
